# Corporate Political Strategies and Discursive Frames of the Gambling Industry in Finland's Gambling Policy Reform

**DOI:** 10.1177/14550725261453262

**Published:** 2026-06-04

**Authors:** Jani Selin

**Affiliations:** 1 3837 Finnish Institute for Health and Welfare, Helsinki, Finland

**Keywords:** corporate political activity, gambling policy, gambling industry, corporate political strategies, discursive framing

## Abstract

**Aims:**

Finnish gambling policy, long based on a state-controlled monopoly, is set to shift to a license-based regime in 2027. The study investigates the Corporate Political Activity (CPA) strategies and discursive frames used by gambling industry actors, comparing their application among actors and phases of the legislative process in the context of Finland's gambling policy reform.

**Methods:**

In total, 36 formal statements were analyzed, including submissions to the public consultation (*n* = 19) and parliamentary hearings (*n* = 17). A deductive content analysis was conducted, based on the previous CPA frameworks.

**Results:**

The results indicate that gambling industry actors proposed alternative policies and sought to prevent certain parts of the legislation from being enacted. Discursive framings shifted throughout the legislative process: legal rights dominated consultations, while unintended effects, especially increased unlicensed gambling, became more salient in parliamentary hearings. Clear differences emerged among actors. International actors prioritized policy substitution and framed the proposed legislation as excessive and harmful to their legal rights, whereas the state monopoly operator did not prevent enactment of regulations. A further difference concerned proposals to replace some harm-reduction measures with more restrictive alternatives.

**Conclusions:**

The growing influence of the gambling industry may risk deprioritizing the prevention and reduction of gambling harm in future gambling policy.

## Introduction

For decades, Finnish gambling policy has been based on state-controlled monopoly. This monopoly regime in the mainland Finland is scheduled to be dismantled in 2027, when online casino gambling and sports betting will be opened to licensed operators, while lottery games and electronic gambling machines will remain under the state monopoly. The whole process with the official aim of reducing unlicensed online gambling was initiated by the gambling industry, the state-owned monopoly company (Thompson, 2025). Compared with the existing monopoly regime, the new gambling legislation is notably more liberal from a public health perspective because it includes the removal of restrictions on the marketing and supply of online casino games (Gambling Act 10/2026). This transition provides a rare opportunity to examine how gambling industry actors have sought to influence policymaking in a context of regulatory liberalization.

The Finnish case is situated within a broader body of research on how addiction industries, such as those producing tobacco, alcohol, and gambling products, have been recognized for their efforts to shape public policy in ways that support their commercial interests (De Lacy-Vawdon et al., 2023). The political engagement of the tobacco and alcohol industries, in particular, has been extensively documented, demonstrating how these sectors seek to influence regulatory frameworks, public discourse, and scientific debate (Martino et al., 2017; Savell et al., 2014).

As the gambling industry expands globally, driven by online gambling across jurisdictions, questions have emerged about the nature and extent of its involvement in policy-making processes (Wardle et al., 2024). Similar to other addiction industries, the gambling sector seeks to influence public policy through a range of strategies: maintaining self-regulation and resisting stronger government regulation; engaging in lobbying and political donations; fostering the movement of personnel between industry and government; cultivating relationships with policymakers and other stakeholders; framing gambling harms as affecting only a small “minority” of individuals and therefore not attributable to specific products; emphasizing individual responsibility; and promoting narrowly targeted harm-reduction measures instead of more effective population-level interventions (Bhuptani et al., 2023; De Lacy-Vawdon et al., 2023; Hancock et al., 2018; Petticrew et al., 2017).

The political influence of the gambling industry has been examined from multiple perspectives, including corporate political activity (Bhuptani et al., 2023; Hancock et al., 2018; Sama & Hiilamo, 2024, 2025), the corporate power and health framework (De Lacy-Vawdon et al., 2023), conceptual analyses (Petticrew et al., 2017), and agnogenic practices (Van Schalkwyk et al., 2024).

The most widely adopted and empirically verified approach for the analysis of the industry political influence is the Corporate Political Activity (CPA) framework, originally developed by Hillman and Hitt (1999) and later developed and expanded by Savell et al. (2014).

The original framework conceptualized corporate political activity as one side of an exchange relationship in which corporations provide policymakers with support and information in return for policy influence. It identified three main strategies for influencing policy, information, financial incentives, and constituency building, each comprising multiple tactics (Hillman & Hitt, 1999).

Savell et al. (2014) extended this model by identifying additional strategies and tactics used by the tobacco industry, and by introducing a new analytical dimension: discursive frames, that is, the interpretive schemata consisting of individual arguments (reasons given for opposing or supporting a policy) through which policy issues are defined and understood. This verified and adaptable framework provides a robust basis for examining gambling industry political activities.

Three studies have applied the CPA framework with an exclusive focus on the gambling industry were identified (Hancock et al., 2018; Sama & Hiilamo, 2024, 2025). In an Australian study focusing on industry strategies, Hancock et al. (2018) identified corporate social responsibility as a new strategy, while all other strategies from the revised CPA framework were also present in the gambling industry's submissions, supporting its applicability to the gambling context. Two studies conducted mainly in the context of Finland analyzed both the strategies employed by the gambling industry (Sama & Hiilamo, 2024) and the arguments advanced by an industry-affiliated association (Sama & Hiilamo, 2025). The former identified lobbying, forming alliances, promoting alternative policies, and supporting self-regulation as strategies to influence policy, as well as the argument that the monopoly was redundant (Sama & Hiilamo, 2024). Regarding the introduction of a licensing regime in Sweden, the study found that the gambling industry influenced policy-making by lobbying and promoting self-regulatory policies (Sama & Hiilamo, 2024). The latter study found that the benefits of introducing a license-based regime, in place of the existing monopoly regime, were a cross-cutting theme in the arguments identified in the social media posts of an industry-affiliated association during the 3-month period investigated (Sama & Hiilamo, 2025).

This study contributes to the existing CPA literature on gambling industry political activity in four ways. First, unlike previous studies, it adopts a comparative approach to examine differences between industry actors in their use of strategies. Prior research has demonstrated variation in the corporate social responsibility practices and policy views of the Nordic gambling operators (González Díaz et al., 2024; Selin, 2024). Second, insofar as is currently known, the use of gambling operators’ corporate political activities across the different stages of the legislative process has not been the focus of prior studies. This study addresses that gap through a longitudinal design, comparing industry submissions on two stages of the legislative process. Third, building on previous research on Finnish gambling policy, the paper advances understanding of industry strategic action in a context where the process involves removing restrictions rather than introducing them, an underexamined situation in the existing literature. Fourth, as suggested by Hancock et al. (2018), there is a need for studies that combine analyses of both strategies and frames, as this provides a more complete understanding of how gambling industry actors seek to shape policy.

To address these gaps in the literature, the present study examines the corporate political activity strategies and discursive frames adopted by gambling industry actors in the context of Finland's reform of gambling legislation. Specifically, it investigates the range of strategies and frames these actors employ, how the strategies and frames differ among actors, and whether the use of strategies and frames changes during two phases of the legislative process.

## Methods

### Data

Reflecting Finland's commitment to participatory democracy, the legislative process in Finland usually includes several opportunities for stakeholder involvement. These typically consist of a public consultation phase, during which stakeholders may comment on a draft government proposal, followed by parliamentary committee hearings on the final proposal before the law is enacted. Parliamentary committees operate autonomously in selecting experts, including academics, public authorities, civil society representatives, and industry stakeholders. As several committees are often involved, the same experts may be invited to multiple hearings. The inclusion of stakeholders in this process aligns with Hillman and Hitt's (1999) conceptualization of corporate political activity as one side of an exchange relationship in which corporations provide policymakers with support and information in return for policy influence. In the reform of the gambling legislation, 126 statements were submitted during the public consultation on the draft government proposal (Ministry of the Interior, 2024), and 144 during the parliamentary hearings where the final government proposal (Hallituksen esitys, 2025) was considered.

The material comprised 36 statements from gambling industry actors (*N* = 19), submitted during the 2024 public consultation (*n* = 19) and the 2025 parliamentary hearings (*n* = 17). Thirteen actors participated only in the consultation phase, and six submitted in both phases, with some appearing at multiple committees (Table 1). The data are publicly available on the websites of the Ministry of the Interior and the Parliament (Table 1). In total, the corpus comprised approximately 73,000 words. The length of individual statements ranged from 88 to 6,469 words. Submissions to the consultation were generally longer (*M* = 2,758 words) than those to the hearings (*M* = 1,217 words).

**Table 1.** Statements Submitted by Gambling Industry Actors During the Legislative Process.

Actor (Year)	Field of Activity
Aid Dice (2024)	Supplier
AB Trav och Galopp (ATG) (2024)	Operator
Betsson Group (2024)	Operator
European Gaming and Betting Association (EGBA) (2024, 2025)	Trade association
Evolution Gaming (2024)	Supplier
Finnish Gambling Association (2024, 2025)	Industry-affiliated association
Finnplay Technologies (2024, 2025)	Supplier
International Betting Integrity Association (IBIA) (2024)	Trade association
Kindred Group (2024)	Operator
Legal Gaming (2024)	Industry-aligned legal firm
Mindway AI (2024)	Service provider
Play’n Go (2024)	Supplier
Playtech (2024)	Supplier
Polar limited (2024)	Supplier
Svenska postkodlotteriet (2024)	Operator
The Finnish Gambling Consultants (2024)	Consultancy
The Gambling Industry Finland (2024, 2025a, 2025b, 2025c, 2025d, 2025e)	Trade association
Veikkaus (2024, 2025a, 2025b, 2025c, 2025d, 2025e)	Operator
Ålands Penningautomatförening (PAF) (2024, 2025a, 2025b, 2025c, 2025d)	Operator

To be included in the dataset, an actor was required to demonstrate a clear and substantive affiliation with the gambling industry. The gambling industry is thus understood here as an ecosystem or “gambling complex” (Markham & Young, 2015) comprising a multiplicity of actors with distinct yet interrelated financial interests in gambling operations. Following the criteria used by Bhuptani et al. (2023), core industry actors, namely gambling operators, service and product suppliers, and trade associations, were included by definition. In addition, a consultancy, a law firm, and an association were classified as industry actors because they clearly maintained enduring and functionally significant relationships with gambling operators, providing support to the industry's collective political activity.

### Procedure

The qualitative design of the study was chosen as qualitative approach allows for detailed contextual analysis of gambling industry political activity. To examine the CPA strategies and discursive frames of the gambling industry in Finland, a deductive content analysis of the manifest meanings was conducted (Elo & Kyngäs, 2008).

The coding frame was derived from previous CPA frameworks (Hancock et al., 2018; Savell et al., 2014; Savell et al., 2016). The coding focused on identifying strategies (comprising specific tactics) and discursive frames (comprising different arguments). To capture potential contextual nuances, a data-driven, inductive approach was also employed to ensure that possible strategies, tactics, frames or arguments specific to the Finnish case were not overlooked. The full coding framework used in the present study is presented in Table 2, and the complete CPA framework that served as the starting point is available in Appendix 1.

**Table 2.** Gambling Industry Strategies, Tactics, Discursive Frames, and Arguments for Influencing Policy Identified in the Finnish Context, Including Descriptions of Frames and Arguments.

Strategy	Tactic
Information (providing or manipulating evidence)	Direct lobbying (statements, meetings and correspondence with policymakers)
	Indirect lobbying (using third parties to lobby on the industry's behalf)
	Shaping the evidence base: Commissioning, writing, or disseminating publications Preparing position papers, technical reports on impacts
	Proposing industry/policymaker collaboration (e.g., via working group)
	Distorting the evidence (claiming that something contentious if factual)
	Selectivity of evidence (resulting in gaps and omissions in evidence)
Constituency building (gaining support of other organizations or members of the public)	External constituency building: Form alliances with other industry sectors/ trade organizations
	Internal constituency building: Collaboration between companies, development of panindustry group, or industry trade association
Policy substitution (proposing or supporting alternative policies)	Promotion of industry self-regulation Promotion of alternative regulatory policy Promotion of non-regulatory initiative
Legal (using the legal system)	Pre-emption (preventing legislation)
Financial incentive (e.g., political donations, financial inducements)	Promise of financial gain for state and civil society
Discursive frame	Argument
Regulatory redundancy frame (arguing that new regulations are unnecessary)	Self-regulation is effective Industry is responsible The industry has a positive impact
Legal frame (arguing that regulations violate legal rights)	Regulations infringe on legal rights: Concerns about trademarks, intellectual, free speech, basic rights Regulations are excessive or disproportionate Regulations interfere with the free market **Lack of clarity in the proposed regulations** **Regulations are too liberal**
Negative unintended consequences frame (arguing that regulations have harmful effects)	Economic arguments: Regulations will impose high compliance costs on companies Regulations will unfairly disadvantage certain businesses or consumers Regulations will negatively impact government revenue Public health arguments: Regulations will have negative public health consequences Other arguments: **Regulations will have negative impact on unlicensed gambling** **Regulations will increase marketing**
Complex policy area frame (arguing that gambling policy is complex as it involves multi-causal social problems)	Industry collaboration with government is beneficial
Insufficient evidence frame (arguing that policy is not based on sound evidence)	There is no evidence that restrictions work

Adapted from the Taxonomies of Savell et al. (2014), Savell et al. (2016), and Hancock et al. (2018), with Newly Identified Arguments Shown in Bold.

The coding was conducted by a single researcher after a thorough reading and rereading of the material. The coding followed an iterative process that involved moving back and forth between reading, applying the codes, and analysis. The paragraph served as the basic unit of coding, and, as a rule, only the explicitly stated main issue or theme within each paragraph was coded. In practice, each paragraph received one strategy code and one discursive frame code. In a few cases, two strategy or two frame codes were applied when a paragraph clearly addressed two distinct themes or issues. The resulting codes were then organized by theme or issue within the broader categories of tactics and arguments. The best available research evidence was used to assess whether industry actors employed the information tactics of *distorting evidence* and *selectivity of evidence* (Hancock et al., 2018) (Table 2).

For the presentation of findings, the relative frequency of codes was categorized for descriptive purposes as very infrequent (< 10%), infrequent (10%–20%), moderately frequent (20%–30%), and frequent (> 30%). These categories were used only to indicate relative emphasis. Results on strategies and frames are presented in order of relative intensity, using illustrative terms (e.g., “central,” “numerous” and “infrequent”) to indicate cross-cutting and less common findings.

The data excerpts in the presentation of the results were selected to illustrate as clearly as possible the specific strategy, tactic, frame or argument under analysis. In addition, the selection aimed to provide as comprehensive a view as possible of the perspectives represented by different industry actors.

No ethical review was required, as the material analyzed consisted solely of publicly available documents.

## Results

### Strategies

Overall, the actors used many different strategies with legal strategy and in particular policy substitution strategy being most often adopted. The relative frequencies of occurrence of the strategies are presented in Table 3.

**Table 3.** Relative Intensity of Strategies.

Strategy	Intensity
Constituency building	●
Information	●●
Legal	●●
Policy substitution	●●●●

● = Very infrequent; ●● = infrequent; ●●● = moderately frequent; ●●●● = frequent.

Note: Only codes with five or more occurrences are shown.

#### Policy Substitution Strategy

The gambling industry frequently relied on a policy substitution strategy, whereby it proposed or supported alternative policies. The *promotion of alternative regulatory policy* was a central tactic employed by all industry actors. A key issue associated with this tactic concerned the draft government proposal's requirement for the impeccability of license applicants (Ministry of the Interior, 2024). In particular, the gambling operator Betsson Group (2024) and The Gambling Industry Association Finland (2024) opposed the proposed impeccability criteria, which stipulated that applicants must not have violated existing legislation by marketing or offering gambling products in Finland within the 5 years preceding the application. Both parties argued that the 5-year assessment period should be shortened.

Another issue that prompted numerous requests to amend the proposed regulations concerned the planned marketing restrictions and the ban on customer bonuses. Across actors, the complete ban on bonuses was opposed, and alternative approaches were suggested. As stated by the Finnish Gambling Association (2024): “… at least allowing welcome bonuses could increase the competitiveness of regulated operators in comparison with unregulated operators” (p. 10). Similarly, industry actors called for either the removal or clarification of the proposed bans on the use of third parties in online marketing, such as affiliates.

Among the proposed measures for the reduction of gambling harm were deposit and loss limits. Opposition arose to the idea that licensee-specific statutory loss limit caps could be issued either by Government Decree or by a Decree of the Ministry of the Interior. Instead, it was suggested that gamblers should be able to determine their own limits. Unlike other operators, the Finnish company Ålands Penningautomatförening (PAF) (2024) repeatedly emphasized that, to protect gamblers, the government should establish by law “a national, unified deposit limit for online gambling that covers all games and all operators within the licensing regime” (p. 2).

Although the *promotion of industry self-regulation* was occasionally employed, it remained a secondary tactic. Its use was largely tied to duty of care practices, particularly in allowing the industry to define the standards and specific indicators of harm to be used. This approach was explicitly endorsed by Veikkaus (2025b), the monopoly operator in the mainland Finland: “The fulfilment of the duty of care must be the responsibility of the companies” (p. 2). *Promotion non-regulatory initiatives* was sporadic.

#### Legal Strategy

The only legal tactic adopted by the gambling industry was *pre-emption*, that is, the prevention of certain provisions of the government proposal of becoming the law. The possibility to issue loss limit caps opposed strongly by many industry actors. For example, the Kindred Group (2024) was against such regulation: “Kindred Group principally opposes the proposed regulatory authority in subsection 2 concerning maximum loss limits” (p. 6). The proposed regulatory authority given to the Ministry of the Interior over game characteristics was similarly opposed (e.g., Finnplay Technologies, 2025).

The industry formed a united front against the regulations prohibiting the use of third parties in marketing and prohibiting the offering of bonuses for customers in the draft government proposal. The European trade organization, European Gaming and Betting Association (EGBA) (2024) opposed the prohibitions in the following way: “We therefore advise against the proposed ban on bonuses which, along with the cumulative effects of the proposed advertising restrictions – particularly the ban … on the use of affiliates – will have negative unintended consequences” (p. 4).

During the consultation, Betsson Group, Kindred Group, and the Gambling Industry Association Finland all opposed the regulation requiring license applicants to demonstrate impeccable conduct during the 5 years preceding the application date, presenting extensive legal arguments in support of their position. Betsson Group (2024) argued that “… retroactive legislation cannot in any event be considered acceptable” (p. 5). EGBA (2025) reiterated these concerns during parliamentary hearings.

#### Information Strategy

Information strategy was infrequently used by the gambling industry actors. *Direct lobbying* was a tactic that characterizes the submission of statements during the consultation as well as the parliamentary hearings. In effect, every submitted statement is an act of direct lobbying. Direct lobbying included general promotion of specific aspects of the new gambling regime or unspecific support for the reform as a whole.

One key theme in the direct lobbying tactic was the protection of the powers and resources of the supervisory authority under the new regulatory framework. A particular emphasis was placed on ensuring that the regulator would have the necessary tools to combat unlicensed online gambling offer. Furthermore, the importance of collaboration between the regulator and the industry was highlighted: “Parliament must require the regulatory authority to engage in dialogue with the industry” (The Gambling Industry Association Finland, 2025a, p. 2).

Another key theme within the direct lobbying tactic concerned the duty of care that the forthcoming legislation was proposed to impose on licensees. The Gambling Industry Association and several suppliers lobbied for industry-developed AI-based behavioural analytics, promoted as tools to detect risky gambling and intervene before serious harm occurred. The industry further sought to define which behavioural indicators such analytics would target: “By analyzing gambling patterns, the operators can identify potential signs of problem gambling … Such analysis includes bet frequency, number of stakes, time, as well as changes in gambling patterns” (Playtech, 2024, p. 5).

Moreover, industry actors lobbied for evaluating the new regime by its channeling rate, the share of online gambling within the regulated market, arguing that effective harm prevention depends on maintaining a high rate.

The tactic of *shaping the evidence base* was evident in the statements analyzed, albeit on only a few occasions. Typically, this strategy appeared when industry-commissioned reports or industry-funded research papers were referenced.

*Distorting the evidence* was very infrequent tactic used by two actors in particular: the Finnish Gambling Association and the law office Legal Gaming. The distortion of evidence was more evident in the context of prevalence of gambling harm, where a research report published by the Finnish Institute for Health and Welfare was incorrectly referenced: “… the prevalence of problem gambling has reached its all-time peak as even 4.2% of the population, that is over 150,000 individuals aged 15 to 74 years, suffer from problem gambling” (Legal Gaming Oy, 2024, p. 11; see also Finnish Gambling Association, 2025). The correct value, however, is that 4.2% of respondents played at a moderate-risk or problem gambling level in 2023 (Grönroos et al., 2024). The statement continued with another distortion, comparing the prevalence rates from the Finnish survey with results from an international comparative study (Tran et al., 2024) that used a different definition of problem gambling, thus making the conclusion, that the prevalence of problem gambling in Finland is three times higher than internationally, factually incorrect.

*Selectivity of evidence* was a tactic adopted by several industry actors, although only very infrequently. Typically, these instances involved actors asserting that the research or experiential evidence they presented unequivocally demonstrated the actual state of affairs or the definite consequences of the proposed regulations.

*Indirect lobbying*, understood here as the use of front groups, was very infrequent tactic; only one law firm and one consultancy met the criteria for indirect lobbying.

#### Constituency Building

Constituency building emerged as a strategy that is difficult to discern from the analyzed textual material. Additional information would be required to enable more reliable interpretations.

One example of potential *external constituency building* tactic can be found in the gambling operator ATG’s (2024) statement, which remarked that many actors within the Finnish equestrian industry shared ATG's view on including horse betting within the licensed competitive market.

The tactic of *internal constituency building* was not explicitly foregrounded in the material; it was mentioned only indirectly when organizations described themselves in their submissions.

#### Financial Incentive Strategy

Financial incentive strategy was very infrequently used, with only a few vague references to state or civil society gains offered in support of a more liberal regulatory approach.

### Discursive Frames

Overall, the legal frame and unintended consequences frame were the most used frames by the gambling industry actors (Table 4). Several arguments within these frames were adopted, especially in connection to the legal frame.

**Table 4.** Relative Intensity of Discursive Frames.

Frame	Intensity
Complex policy	●
Legal	●●●●
Regulatory redundancy	●
Unintended consequences	●●●●

● = Very infrequent; ●● = infrequent; ●●● = moderately frequent; ●●●● = frequent.

Note: Only codes with five or more occurrences are shown.

### Legal Frame

Unsurprisingly, the legal frame was employed extensively in the legislative process analyzed here. The most common argument, not identified in previous studies, within this frame concerned the *lack of clarity in the proposed regulations*. Thematically, these arguments centered on the terminology used in the provisions on marketing, which industry actors considered overly open to interpretation. As stated in one submission: “Overall, the section on the regulation of marketing, the terminology used, and particularly the detailed reasoning does not sufficiently specify which marketing practices are permitted” (Finnish Gambling Association, 2024, p. 13).

Another argument within the legal frame was that the proposed *regulations are excessive or disproportionate*. This argument was mainly associated with the draft government proposal's (Ministry of the Interior, 2024) requirement that license applicants demonstrate impeccable conduct during the 5 years preceding their application. Betsson Group (2024) in particular emphasized this point: “Thus, the proposed provision of the Gambling Act, according to which a mere administrative sanction imposed by the National Police Board would constitute an automatic barrier to obtaining a license and thereby restrict the fundamental right to freedom of trade, is unfounded and clearly disproportionate within the Finnish legal order” (p. 14). Furthermore, the draft government proposal's proposed ban on the use of third parties in marketing was also considered excessive, with some submissions arguing that it would have made sponsorship arrangements extremely difficult.

Within the legal frame, many gambling industry actors argued that the proposed *regulations would interfere with free market*. This argument was almost entirely directed at the position of the existing state monopoly, Veikkaus, in the new regulatory regime. It was claimed that the part of Veikkaus Group operating in the competitive online casino and betting market would gain an unfair advantage by sharing resources, such as profits, customer data, and technology, with the branch operating in the largely land-based monopolistic market. Concerns about potential market distortion were raised in several respects: “Several of the proposed regulations will lead to the protection of the present monopoly operator in the future regime, which will distort fair competition” (The Gambling Industry Association Finland, 2024, p. 22).

Arguments were also presented concerning *infringements of legal rights*. These arguments referred to a range of rights and principles, including fundamental rights, freedom of trade, the principle of legal certainty, the principle of good administration, fundamental freedoms, the Finnish Constitution, and the protection of legitimate expectations. However, mostly alleged infringements related to the requirement that license applicants demonstrate impeccable conduct prior to submitting their application.

#### Negative Unintended Consequences Frame

The negative unintended consequences frame was used extensively. Arguments in this frame focused on the risk that the *regulations will have negative impact on unlicensed gambling*. Industry actors argued that the proposed restrictions or bans on customer bonuses and the use of affiliates in marketing would negatively affect the channeling rate. Playtech (2024), a provider of gambling products for operators, stated that a ban on bonuses would increase unlicensed gambling: “… experiences and research on channeling indicate that a ban on bonuses or major restrictions on campaigns would probably have a negative impact on the channeling rate” (p. 4). Furthermore, many submissions considered regulations introducing player-specific spending limits across the licensed market, licensee-specific loss limits, and regulations on game product characteristics to be detrimental to channeling rate.

The arguments on economic unintended consequences were focused on the insight that provisions on applying licenses as well as the licensing conditions as *regulations will impose high compliance costs on companies*.

There were only a few scattered arguments that *regulations will have negative public health consequences*. Two questioned the logic of placing the most addictive products on the open market while keeping less harmful ones under monopoly control.

#### Regulatory Redundancy Frame, complex Policy Area Frame, and Insufficient Evidence Frame

Overall, arguments aligned with the *regulatory redundancy frame* were seldom employed by the gambling industry. Only a few scattered claims were made, typically emphasizing the industry's positive impact on society, effectiveness of industry self-regulation, or its responsibility. The *complex policy area frame* and *insufficient evidence frame* were adopted only sporadically. The latter was employed to argue that the industry should be more involved in the legislative process or that the regulatory authority should be in dialogue with the licensees when interpreting the law.

### Differences Between the Gambling Industry Actors’ Strategies and use of Frames

Overall, there were clear differences in the strategies chosen by industry actors. The most used approaches were information strategies, policy substitution, and legal strategies, with policy substitution being particularly widespread. The direct lobbying tactic becomes more common in the hearings (Table 5). Several major actors (PAF, Kindred Group, Betsson Group, and Veikkaus) made limited use of information strategies during the consultation phase but adopted them more actively during the parliamentary hearings. The international companies and their industry organization (ATG, Kindred Group, Betsson Group, The Gambling Industry Finland) relied on legal strategies more than other actors, whereas Veikkaus made almost no use of the legal (pre-emption) strategy.

**Table 5.** Relative Intensity of Tactics Used in Submissions During the Consultation and Parliamentary Hearings.

Strategy/tactic	Consultation	Hearings
Information strategy		
Direct lobbying	●	●●●
Shaping the evidence base	●	–
Distorting the evidence	●	●
Selectivity of evidence	●	–
Legal strategy		
Pre-emption	●●	●
Policy substitution strategy		
Promotion of self-regulation	●	–
Promotion of alternative regulatory policy	●●●●	●●●●

● = Very infrequent; ●● = infrequent; ●●● = moderately frequent; ●●●● = frequent.

Note: Only codes with five or more occurrences are shown.

The legal frame and the unintended consequences frame were the most employed across the legislative process. There were clear differences between the two phases of the legislative process (Table 6). During the consultation phase, legal frame arguments concerning the excessiveness of proposed regulations appeared occasionally but were prominent in the submissions by Playtech (2024) and Betsson Group (2024). Similarly, arguments concerning infringements on gambling companies’ legal rights were more prevalent during the consultation phase, with these concerns primarily raised by international companies and their organizations. By contrast, concerns about unclear or ambiguous provisions were less frequently raised in the parliamentary hearings.

In contrast, during the hearings, arguments concerning unlicensed gambling and legal arguments emphasizing overly liberal provisions became more common. The argument that the *regulations are too liberal* was entirely new. PAF, in particular, criticized the proposed spending limits as being too liberal from the perspective of gambling harm, advocating instead for centralized loss limits rather than licensee-specific deposit limits (e.g., Ålands Penningautomatförening, 2025a). The Finnish Gambling Association's (2025) submission to Parliament focused heavily on the negative consequences the proposals would have on unlicensed gambling. In contrast, Veikkaus rarely invoked unlicensed gambling as an unintended consequence in its arguments.

**Table 6.** Relative Intensity of Arguments Adopted in Submissions During the Consultation and Parliamentary Hearings, with Newly Identified Arguments Highlighted.

Frame/argument	Consultation	Hearings
Legal		
Regulations are excessive or disproportionate	●●	–
Regulations infringe on legal rights	●●	●
Regulations interfere with the free market	●	●●
Regulations are too liberal	●	●●
Lack of clarity in the proposed regulations	●●●	●●●
Negative unintended consequences		
Regulations will impose high compliance costs on companies	●	–
Regulations will increase marketing	●	–
Regulations will have negative impact on unlicensed gambling	●●	●●●●
Regulations will have negative public health consequences	●	–

● = Very infrequent; ●● = infrequent; ●●● = moderately frequent; ●●●● = frequent.

Note: Only codes with five or more occurrences are shown.

## Discussion

The present study investigated the corporate political activity strategies and discursive frames used by gambling industry actors, comparing their application across actors and phases of the legislative process. The results indicate that gambling industry actors most frequently employed policy substitution and legal strategies with the legal and unintended consequences frames being the primary frames. Clear differences emerged among actors. Betsson Group and The Gambling Industry Association Finland prioritized policy substitution and legal strategies, whereas the state monopoly Veikkaus avoided the legal tactic pre-emption altogether and rarely invoked unlicensed gambling as an unintended consequence. Betsson Group also emphasized the frame portraying regulations as excessive or disproportionate. PAF was an exception, adopting the policy substitution strategy while arguing, within the legal frame, that spending limit regulations were overly liberal.

It is noteworthy that the CPA framework assumes industry actors primarily oppose regulatory restrictions. However, this case study illustrates that, under certain conditions, industry actors may support proposed legislation or even advocate for additional restrictions, as exemplified by PAF. The approach by PAF may reflect its earlier strategic decision to impose mandatory annual loss limits on all customers. Moreover, the current monopoly operator, Veikkaus, adopted a markedly different approach compared to international operators. Unlike the international operators, Veikkaus did not focus on arguing that the proposals would lead to unintended consequences such as an increase in unlicensed gambling, nor did it employ the legal pre-emption tactic. One possible reason for this is that Veikkaus strongly supported the reform and its framing as a solution to the policy problem of unlicensed gambling in Finland (Thompson, 2025). These differences in interests suggest that the gambling industry is heterogeneous and should be considered in the continued development of the CPA framework and other research.

Betsson Group and others’ emphasis on policy substitution and the legal tactic of pre-emption likely reflected the National Police Board's 2023 marketing ban on a Betsson subsidiary for illegal gambling advertising, imposed with a conditional fine that could have jeopardized the company's licensing eligibility in 2027. Ultimately, the final government proposal and the Gambling Act shortened the required period of impeccable conduct from 5 years to 2 years. This can be interpreted as an example of successful policy influence, similar to the findings of Sama and Hiilamo (2024) regarding Sweden's transition to a license-based regime.

In contrast to previous research (Hancock et al., 2018; Sama & Hiilamo, 2024), three strategies, financial incentives, constituency building, and corporate social responsibility, were absent or rarely used, likely because they occur outside the publicly submitted statements analyzed here. Similarly, the tactics of promoting non-regulatory initiatives and self-regulation, as well as several discursive frames (insufficient evidence, complex policy area, and regulatory redundancy), were used rarely, despite being common in earlier studies (Bhuptani et al., 2023; Hancock et al., 2018; Petticrew et al., 2017; Sama & Hiilamo, 2024). One possible explanation for their limited use is that the Finnish legislative reform centers on removing restrictions, which offers potential financial benefits for the industry. This suggests that the gambling industry engages in strategic adaptation when influencing policy across different contexts, a finding that may have relevance beyond the Finnish case.

There was notable overlap between certain tactics and discursive frames. Similar findings have been made in previous CPA research, with Savell et al. (2014) suggesting that such overlaps indicate that industry tactics and arguments can be mutually reinforcing. In this study, the legal tactic of pre-emption frequently co-occurred with the promotion of alternative regulatory policies, implying that preventing legislation was the primary objective, while advocating alternative regulation served as a secondary one. Both tactics also overlapped with the legal and unintended consequences frames, suggesting that proposals for alternative regulations were often justified by claims that proposed overly restrictive measures would cause unlicensed gambling.

The use of arguments concerning the threat of unlicensed gambling illustrates the malleability of the industry's tactics and arguments because many of those raising such concerns are currently themselves unregulated operators in Finland. This issue was not directly addressed, but the international gambling operators could not submit statements directly in the hearings and were represented instead by the Gambling Industry Association Finland and EGBA, indicating that their access to the legislative process was partially restricted.

Thematically, the submissions discussed extensively the provisions concerning marketing and the offering of bonuses to gamblers. There was also clear opposition to provisions allowing the issuance of decrees on spending limits and game characteristics. These focal points reveal the industry's core interests because they represent regulatory measures that actors sought either to pre-empt or to replace with less restrictive alternatives. Notably, bonuses were permitted in the final government proposal and the enacted Gambling Act. This dynamic underscores the fundamental tension between public health objectives and the financial imperatives of the gambling industry (Selin, 2024).

As with all research, this study has certain limitations. First, the data analyzed provides only a partial view of the gambling industry's activities. Much of the industry's political activities occurs behind closed doors and would require access to alternative sources, such as internal documents or interviews with industry representatives. Second, the transferability of the findings may be limited by the specificity of the legislative case examined. Nevertheless, the results are broadly consistent with previous studies employing the CPA framework, supporting the transferability of the results. Third, the basic unit of coding was the paragraph, and a more fine-grained approach may have revealed additional strategies and frames. However, it is unlikely that this would have substantial impact on the overall results. Finally, systematic comparison of industry operators by type was not conducted, because most actors submitted only during the consultation phase, which would have complicated phase comparisons.

To conclude, the gambling industry is heterogeneous, and its actors adapt their political activities to the specific context in which they operate. Nevertheless, the findings indicate that many policies aimed at effectively reducing and preventing gambling harm are often opposed by the industry. It is also possible that gambling industry actors will continue their political activities in Finland and seek to influence gambling policy. The transition to a license-based regime represents a major shift in Finnish gambling policy, in which the reduction and prevention of gambling harm are likely to be deprioritized. If the reduction and prevention of gambling harm are deprioritized and the power and influence of the gambling industry increase, then this development could, from a public-health perspective, lead to a permanent weakening of efforts to prevent gambling harm.

## Supplemental Material

sj-docx-1-nad-10.1177_14550725261453262 - Supplemental material for Corporate Political Strategies and Discursive Frames of the Gambling Industry in Finland's Gambling Policy Reform Supplemental material, sj-docx-1-nad-10.1177_14550725261453262 for Corporate Political Strategies and Discursive Frames of the Gambling Industry in Finland's Gambling Policy Reform by Jani Selin in Nordic Studies on Alcohol and Drugs

## Data Availability

The data supporting this study are publicly available on the websites of the Ministry of the Interior, Finland, and the Parliament of Finland. These sources are cited within the article.
