# Supplementary material for: Corporate Political Strategies and Discursive Frames of the Gambling Industry in Finland's Gambling Policy Reform
Source: Nordisk Alkohol Nark. 2026 Jun 4:14550725261453262. Online ahead of print. doi: 10.1177/14550725261453262 (PMC13236718; doi:10.1177/14550725261453262)
Supplement: sj-docx-1-nad-10.1177_14550725261453262 - Supplemental material for Corporate Political Strategies and Discursive Frames of the Gambling Industry in Finland's Gambling Policy Reform [file sj-docx-1-nad-10.1177_14550725261453262.docx]

**Appendix 1.**

Table 1. Gambling industry strategies and frames for influencing policy, based on the taxonomies of Savell et al. (2014), Savell et al. (2016), and Hancock et al. (2018).

| **Strategy** | **Tactic** |
| --- | --- |
| Information (providing or manipulating evidence) | Direct lobbying (meetings and correspondence with legislators/policymakers) |
|  | Indirect lobbying (using third parties, including front groups, to lobby on the industry’s behalf) |
|  | Shaping the evidence base:   - Commissioning, writing (or ghost writing), or disseminating research/publications - Preparing position papers, technical reports or data on impacts |
|  | Establishing industry/policymaker collaboration (e.g. via working group, technical group, advisory group). |
|  | Distorting the evidence (claiming that something contentious if factual). |
|  | Selectivity of evidence resulting in gaps and omissions in evidence |
| Constituency building (gaining support of other sectors, organizations, or members of the public) | External constituency building   - Form alliances with and mobilise other industry sectors/ business/trade organisations - Media advocacy (press releases, publicity campaigns, public hearings, interviews) - Form alliances with or mobilise unions/civil society organisations/ consumers/employees/the public - Creation of front groups or astroturf organisations |
|  | Internal constituency building   - Collaboration between companies/development of panindustry group or industry trade association. |
| Policy substitution (proposing or supporting alternative policies) | - Promote voluntary code/self-regulation - Promote alternative regulatory policy - Promote non-regulatory initiative |
| Legal (using the legal system) | - Pre-emption - Using litigation/threat of legal action |
| Constituency fragmentation and destabilization (weakening the opposing actors) | Preventing the emergence of, neutralizing and/or discrediting potential opponents |
| Financial Incentive (e.g. political donations, financial inducements) | - Providing current or offering future employment to those in influential role - Gifts, entertainment or other direct financial inducement |
| Corporate social responsibility (promotion of self-imposed responsibility) | - Industry commitment to ‘responsible’ operations - Pre-emptive industry establishment of internal CSR units/practices |
| **Frame** | **Argument** |
| Regulatory redundancy frame (arguing that new regulations are unnecessary) | - Self-regulation is effective.   - The industry adheres to voluntary codes, which are working well or are better than formal regulation. - Marketing targets only legal-age consumers.   - The industry actively opposes marketing to minors. - Existing regulation is sufficient. (ei)   - Current regulations are adequate but require better enforcement. - Industry is responsible.   - The industry claims to promote responsible consumption. - Focus should be on individual responsibility. - The industry has a positive impact.   - The industry contributes to the economy and community. |
| Legal frame (arguing that regulations violate legal rights) | - Regulations infringe on legal rights.   - Cites concerns about trademarks, intellectual, free speech, or international trade agreements, basic rights). - Regulations are excessive or disproportionate.   - Policies go beyond what is necessary. - Regulations interfere with the free market.   - Restrictions violate economic principles. |
| Negative unintended consequences frame (arguing that regulations have harmful effects) | *Economic*   - Regulations will impose high compliance costs or burden on companies. - Regulations will cause financial/job losses in the industry. - Regulations will unfairly disadvantage certain businesses or consumers. - Regulations will negatively impact government revenue.   - Taxes from gambling sales will decrease. - Regulations will result in job losses in associated industries.   *Public health*   - Regulations will have negative public health consequences.   *Other*   - Regulation could have other negative unintended consequences. |
| Complex policy area frame (arguing that gambling policy is complex because it involves addressing multi-causal social problems) | - The issue is too complex for simple regulation.   - Gambling harm is caused by multiple factors. - Industry collaboration with government is beneficial.   - The industry should be a policy partner rather than be regulated. - Public health advocates and policymakers are authoritarian.   - Those pushing for stricter policies are imposing unnecessary restrictions. |
| Insufficient Evidence Frame (arguing that policy is not based on sound evidence) | - There is no evidence that restrictions work. - There is insufficient evidence linking gambling to harm. |
